# Cardiac magnetic resonance imaging reveals myocardial fibrosis and systolic dysfunction in mitochondrial trifunctional protein-deficient mice

**DOI:** 10.1242/dmm.052921

**Published:** 2026-09-25

**Authors:** Eduardo Vieira Neto, Shakuntala Basu, Thomas J. Becker-Szurszewski, Sean Hartwick, Kristina E. Schwab, Yijen Wu, Jerry Vockley

**Affiliations:** ^1^ Department of Pediatrics, Genetic and Genomic Medicine Division, UPMC Children's Hospital of Pittsburgh, University of Pittsburgh, Pittsburgh, PA 15224, USA; ^2^ Children's Neuroscience Institute, Department of Pediatrics, School of Medicine, University of Pittsburgh, Pittsburgh, PA 15224, USA; ^3^ Division of Neurology and Child Development, School of Medicine, University of Pittsburgh, Pittsburgh, PA 15224, USA; ^4^ Rangos Research Center Animal Imaging Core, School of Medicine, University of Pittsburgh, Pittsburgh, PA 15224, USA; ^5^ Department of Human Genetics, School of Public Health, Center for Rare Disease Therapy, UPMC Children's Hospital of Pittsburgh, University of Pittsburgh, Pittsburgh, PA 15261, USA

**Keywords:** Mitochondrial trifunctional protein deficiency, Cardiac magnetic resonance imaging, Myocardial fibrosis, Fatty acid oxidation disorders, Cardiomyopathy in mouse models

## Abstract

Cardiomyopathy is an important manifestation in patients with fatty acid oxidation disorders and represents a major cause of morbidity and early mortality in mitochondrial trifunctional protein (TFP) deficiency. Although a mouse model carrying the TFP β-subunit p.Met404Lys mutation (βTFP-deficient mice) has been described, cardiac involvement in this model has not been systematically characterized. Here, we combined cardiac histology and multiparametric cardiac magnetic resonance imaging (CMR) to define myocardial structure, function and tissue characteristics in this mouse model. Histological analysis with automated whole-slide collagen quantification revealed myocardial fibrosis with collagen deposition in mutant hearts, and CMR demonstrated increased myocardial extracellular volume in both male and female homozygous mutants. Homozygous males showed reduced ejection fraction, impaired systolic strain and increased left ventricular end-systolic volume, indicating systolic dysfunction. Male mice were more severely affected than females and exhibited reduced survival. Together, these findings demonstrate that βTFP-deficient mice develop fibrotic cardiomyopathy with systolic dysfunction, reproducing important cardiac features observed in human TFP deficiency. This work establishes the model as a relevant platform for investigating disease mechanisms and therapeutic strategies for cardiomyopathy in TFP deficiency.

## Teaser

**Summary:** This study characterizes the cardiomyopathy developed by a mouse model carrying a mutation in the β-subunit of mitochondrial trifunctional protein through multi-parametric cardiac magnetic resonance imaging and histology, validating a relevant disease model.

## INTRODUCTION

Mitochondrial fatty acid β-oxidation (FAO) is a critical source of cellular energy during fasting and physiological stress (Merritt et al., 2018). Long-chain fatty acids are mobilized from adipose tissue, transported into mitochondria and sequentially degraded by β-oxidation. The final three enzymatic steps of long-chain FAO are catalyzed by mitochondrial trifunctional protein (TFP), a heterotetramer composed of two α-subunits encoded by *HADHA* and two β-subunits encoded by *HADHB*. The TFP α-subunit contains long-chain enoyl-CoA hydratase and long-chain 3-hydroxyacyl-CoA dehydrogenase (LCHAD) activities, whereas the TFP β-subunit contains the long-chain 3-ketoacyl-CoA thiolase activity (Xia et al., 2019). Pathogenic variants in *HADHA* and *HADHB* typically result in reduced activity of both α- and β-subunits, leading to a generalized TFP deficiency phenotype, whereas a recurrent *HADHA* c.1528G>C missense pathogenic variant accounts for about 87% of the alleles in isolated LCHAD deficiency (den Boer et al., 2002; IJlst et al., 1996).

Cardiomyopathy has been described in 47% of a large cohort of children with FAO disorders (FAODs) before the implementation of newborn screening (Baruteau et al., 2013, 2014). Dilated and hypertrophic cardiomyopathy have been reported across multiple other long-chain FAODs (LC-FAODs), including deficiencies of very-long-chain acyl­CoA dehydrogenase (VLCAD, also known as ACADVL), carnitine acylcarnitine translocase (CACT, also known as SLC25A20) and carnitine palmitoyltransferase 2 (CPT2) (Baruteau et al., 2013, 2014; Houten et al., 2016). Cardiomyopathy is a significant cause of morbidity and early mortality, especially in TFP/LCHAD deficiency, even in patients identified by newborn screening (Adeva-Andany et al., 2019; Houten et al., 2016; Merritt et al., 2018). Although cardiomyopathy has been reported in patients with both *HADHA* and *HADHB* variants, it is particularly frequent in isolated LCHAD deficiency (den Boer et al., 2002; Kwiatkowska et al., 2017; Stenson et al., 2020).

The C57BL/6J-*Hadhb*^m1Yt^ βTFP-deficient mouse model, carrying a missense mutation in exon 14 (c.1210T>A;p.Met404Lys) of *Hadhb*, was generated using *N*-ethyl-*N*-nitrosourea mutagenesis (Kao et al., 2006). The study reported the original phenotypic characterization, including reduced lifespan due to sudden death, cardiac fibrosis and arrhythmia, and hepatic steatosis. We expanded this phenotype to include cold intolerance after fasting and reduced treadmill exercise endurance (Vieira Neto et al., 2026). Although several mouse models of LC-FAODs have provided important insights into metabolic stress responses and cardiac vulnerability, detailed *in vivo* characterization of cardiac structure, function and tissue composition remains limited (Babcock et al., 2024; Spiekerkoetter and Wood, 2010). Cardiac magnetic resonance imaging (CMR) enables comprehensive, non-invasive and reproducible assessment of myocardial structure, ventricular function and tissue characteristics, and is increasingly considered the reference standard for cardiomyopathy phenotyping in both clinical and preclinical settings (Wu, 2021). In this study, we used comprehensive multi-parametric CMR with established protocols to evaluate cardiac structure, function and tissue composition in βTFP-deficient mice.

## RESULTS

### βTFP-deficient mice exhibit myocardial interstitial fibrosis

Myocardial interstitial fibrosis was observed in all eight homozygous βTFP-deficient mice aged 4-7 months from a separate histological cohort. Picrosirius Red (PSR) staining demonstrated increased collagen deposition within the myocardial interstitium and surrounding intramyocardial vessels. Representative low-magnification (5×) and higher-magnification (20×) images from the mouse exhibiting the highest collagen fraction illustrate the histopathological appearance of myocardial collagen deposition together with the corresponding markup images generated using HALO software (Fig. 1A-D).

**Fig. 1.**
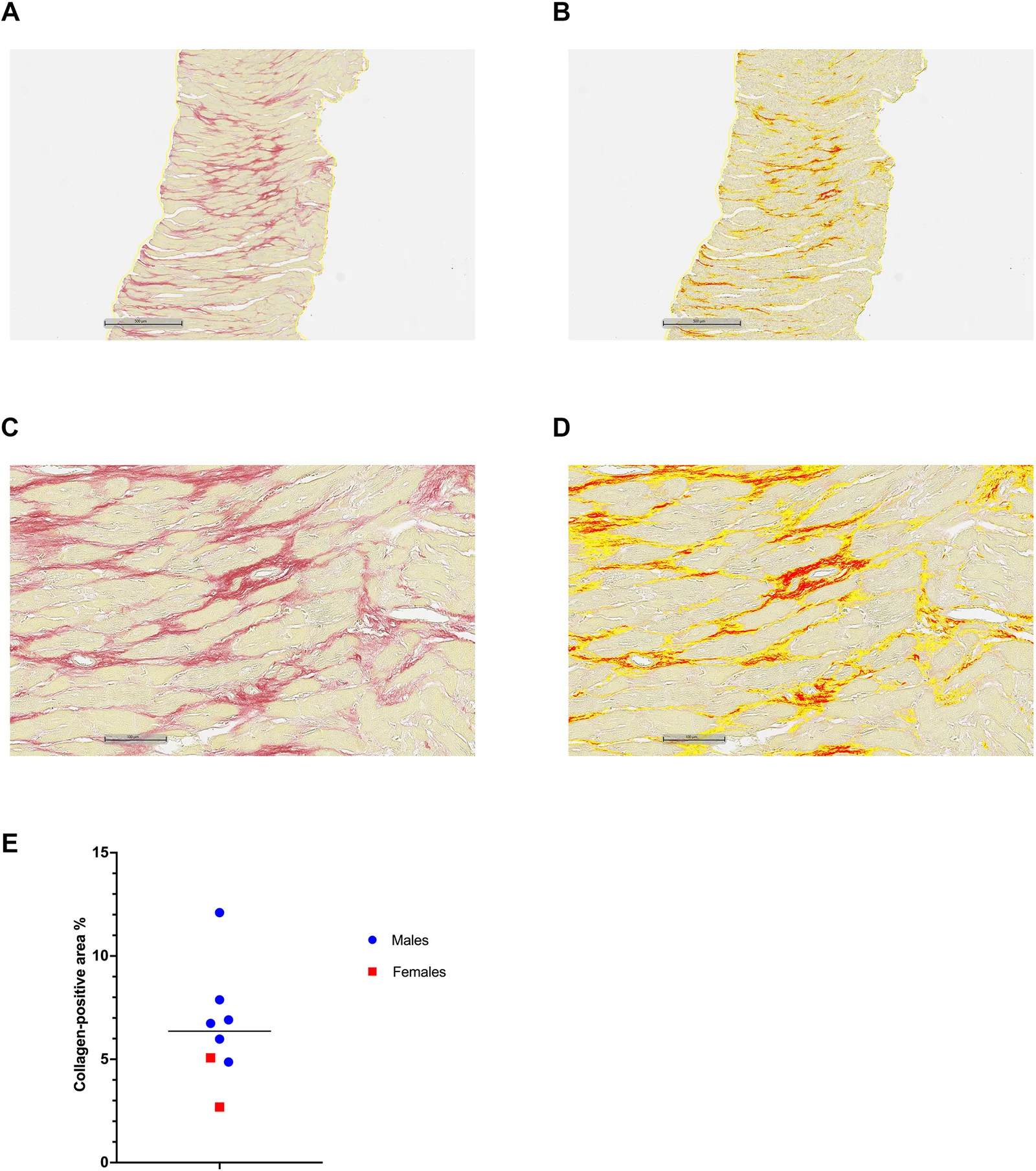
**Myocardial interstitial fibrosis in a homozygous βTFP-deficient mouse with the highest collagen fraction.** Representative Picrosirius Red (PSR)-stained myocardial images from the homozygous βTFP-deficient mouse exhibiting the highest collagen fraction. (A) Low-magnification (5×) PSR image. (B) Corresponding HALO-generated markup image of the same field, showing collagen-positive areas with a yellow-to-red gradient representing increasing PSR staining intensity. (C) Higher-magnification (20×) PSR image. (D) Corresponding HALO-generated markup image of the same field at higher magnification, showing collagen-positive areas with a yellow-to-red gradient representing increasing PSR staining intensity. Scale bars: 500 μm (A,B); 100 μm (C,D). (E) Quantitative collagen fraction determined by HALO analysis of PSR-stained myocardial sections from a separate cohort of homozygous βTFP-deficient mice (*n*=8; six males and two females). Each symbol represents one animal. The horizontal line indicates the median collagen fraction.

Automated whole-slide image analysis using HALO confirmed the presence of myocardial fibrosis in all animals. The quantitative collagen fraction ranged from 2.69% to 12.10%, with the highest value observed in the representative severely affected mouse (Fig. 1E).

### Cine CMR demonstrates impaired systolic emptying in βTFP-deficient mice

Representative long-axis and short-axis cine CMR images illustrate global systolic function in wild-type and βTFP-deficient mice (Fig. 2). In mutant hearts, the left ventricle shows incomplete systolic emptying, with visibly increased end-systolic blood volume, compared with that in sex-matched wild-type controls. These differences are evident in both imaging planes and are more pronounced in male βTFP-deficient mice. Consistent with these observations, quantitative analysis in a larger cohort demonstrated reduced left ventricular ejection fraction (LVEF) in both male and female mutants, with greater impairment in males, accompanied by increased left ventricular end-systolic volume (LVESV) (Fig. 3, Table 1).

**Fig. 2.**
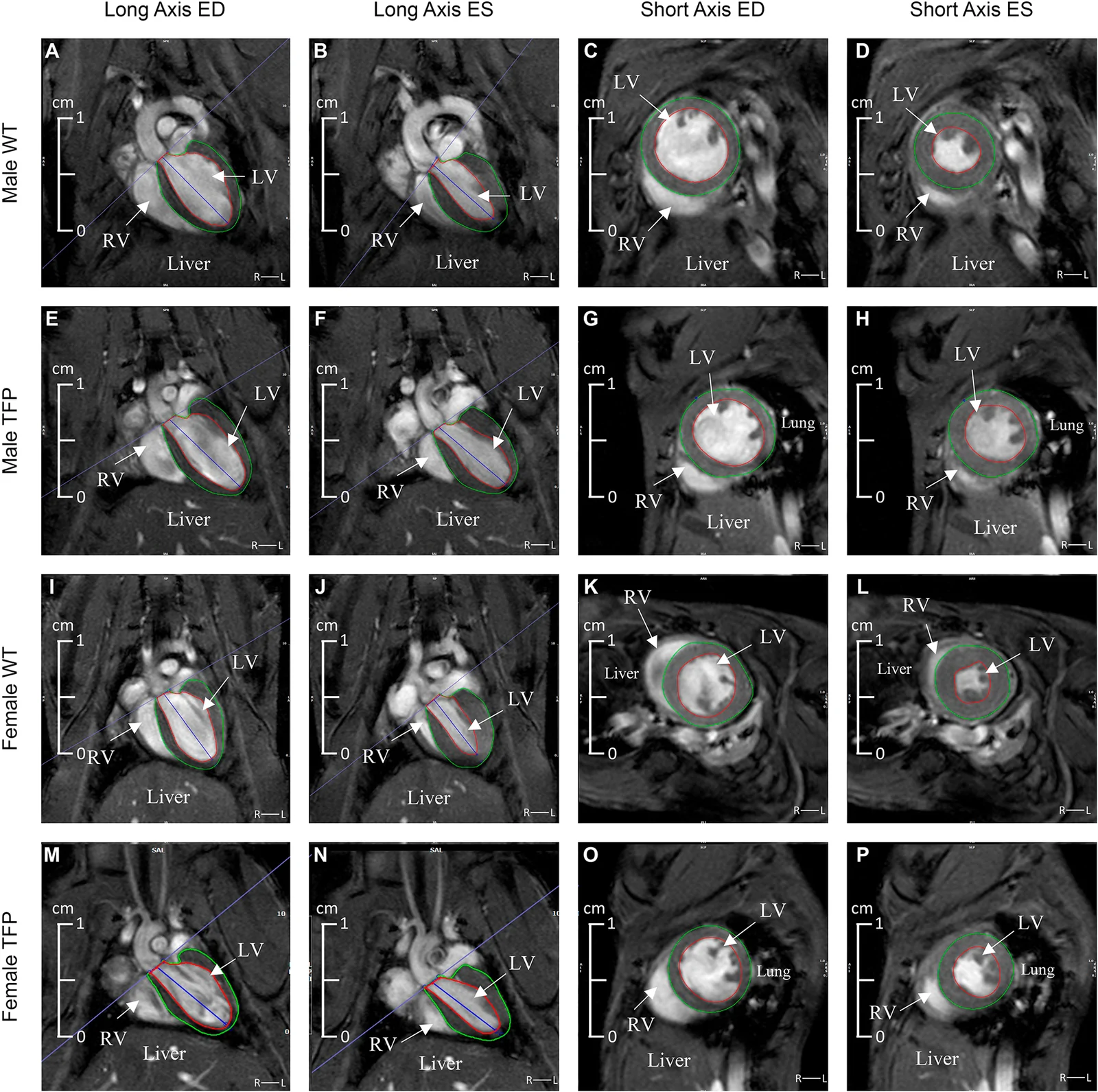
**Representative cine magnetic resonance images illustrating global systolic function in wild-type and βTFP-deficient mice.** (A-P) Long-axis and short-axis cine magnetic resonance images are shown for a wild-type (WT) male (A-D), a βTFP-deficient male (E-H), a WT female (I-L) and a βTFP-deficient female (M-P). For each genotype and sex, end-diastolic (ED) and end-systolic (ES) frames are displayed side by side, with long-axis views on the left and short-axis views on the right. LV, left ventricle; RV, right ventricle. Arrows indicate left (L) and right (R) sides of the body. Green outlines show the external epicardial border; red outlines show the internal endocardial border. Scale bars on the left of each panel are in cm.

**Fig. 3.**
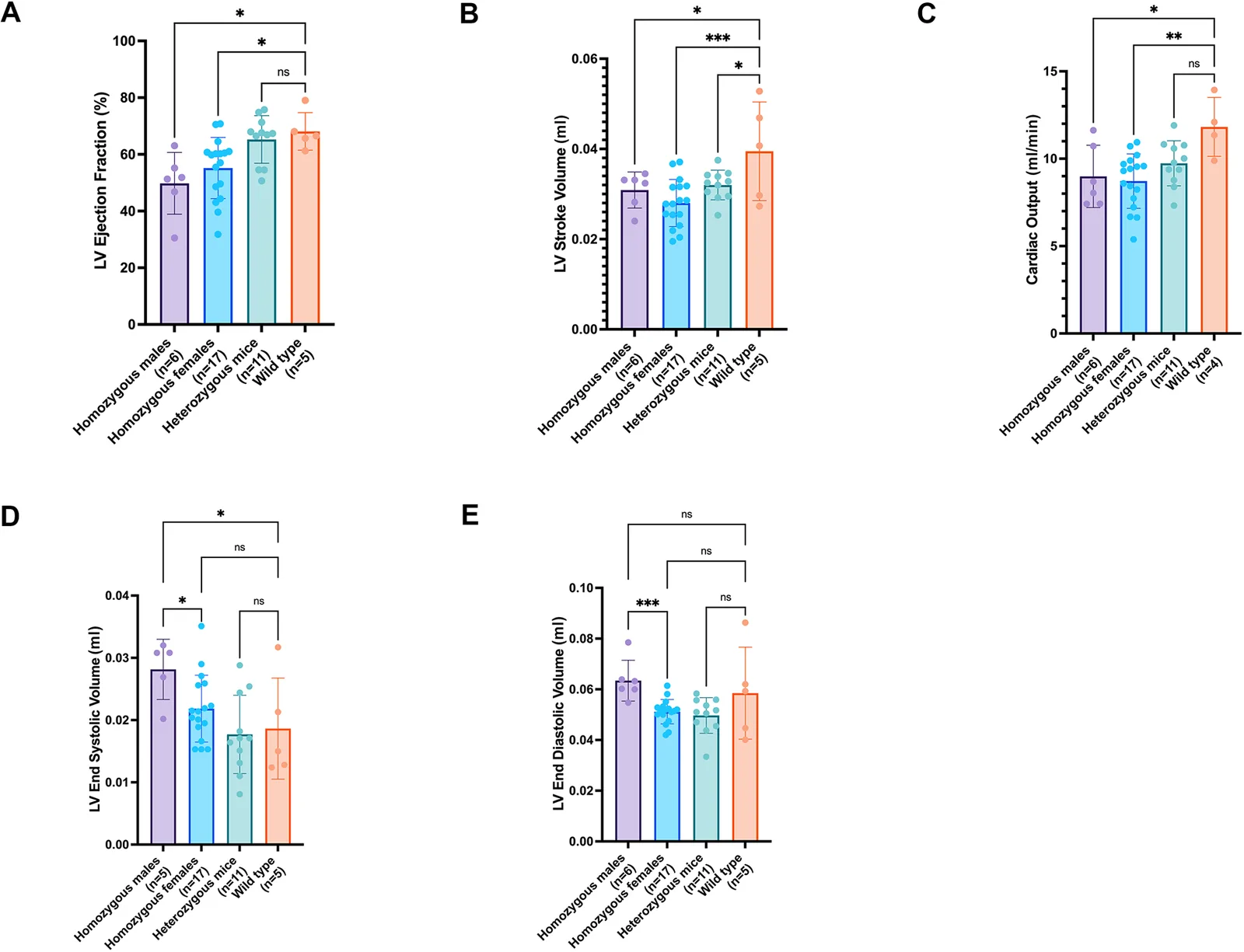
**Global systolic function and ventricular volumes in wild-type C57BL/6J mice, and heterozygous and homozygous βTFP-deficient mice.** (A-E) Quantification of left ventricular (LV) ejection fraction (A), LV stroke volume (B), cardiac output (CO; C), LV end-systolic volume (LVESV; D) and LV end-diastolic volume (LVEDV; E). Homozygous βTFP-deficient mice are shown separately by sex; wild-type and heterozygous mice are shown as combined groups. Sample sizes are indicated in each panel. Differences in sample size reflect exclusion of individual outliers identified by Grubbs' test (α=0.05), as described in the Materials and Methods. Data are presented as scatter plots with bars indicating mean±s.d. Statistical comparisons among groups were performed using one-way ANOVA followed by Dunnett's, Šídák's or Tukey's multiple comparisons testing, as appropriate, with wild-type mice as the reference group. Direct comparisons between homozygous male and female mice are also shown for LVESV (D) and LVEDV (E) and were performed using unpaired two-tailed *t*-tests. ns, not significant, *P*>0.05; *P≤0.05; **P≤0.01; ***P≤0.001.

**Table 1.** CMR-derived global systolic function parameters

Mouse group	LVEF (%)	SV (μl)	CO (ml/min)	LVESV (ml)	LVEDV (ml)
Wild type	68.1±6.6	39.5±10.9	11.82±1.7	0.019±0.008	0.058±0.018
Homozygous males	49.8±10.9 (0.0114)	30.9±4.0 (0.0381)	9.0±1.8 (0.0169)	0.028±0.005 (0.0496)	0.063±0.008 (0.6045)
Homozygous females	55.2±10.8 (0.0397)	28.0±5.2 (0.0008)	8.7±1.6 (0.0023)	0.022±0.005 (0.6578)	0.051±0.005 (0.2049)
Heterozygous	65.3±8.4 (0.9327)	32.0±3.3 (0.0435)	9.7±1.3 (0.0581)	0.018±0.006 (0.9882)	0.050±0.007 (0.1345)

CMR, cardiac magnetic resonance imaging; LVEF, left ventricular ejection fraction; SV, stroke volume; CO, cardiac output; LVESV, left ventricular end-systolic volume; LVEDV, left ventricular end-diastolic volume. Groups: homozygous βTFP-deficient mice are shown separately by sex; wild-type and heterozygous βTFP-deficient mice are shown as combined groups. Sample sizes: wild type, *n*=5; heterozygous, *n*=11; homozygous males, *n*=6; homozygous females, *n*=17; with the following exceptions. For cardiac output, one wild-type outlier identified by Grubbs' test (α=0.05) was excluded (*n*=4). For LVESV, one homozygous male outlier identified by Grubbs' test was excluded (*n*=5). Statistics: data are presented as mean±s.d., with *P*-values in parentheses. Statistical comparisons were performed using one-way ANOVA followed by multiple comparisons tests (Dunnett's or Šídák's, as appropriate) with wild-type mice as the reference group.

### Impaired systolic function in βTFP-deficient mice

Global systolic function was assessed using LVEF, stroke volume (SV) and cardiac output (CO). One-way ANOVA with Šídák's post hoc testing demonstrated reduced LVEF in homozygous βTFP-deficient mice of both sexes compared with that in wild-type controls (Fig. 3, Table 1). Multivariable linear regression analysis confirmed this genotype-dependent reduction after adjustment for age and sex, although age showed a modest independent association with LVEF (Table S1).

For SV and CO, unadjusted group comparisons also indicated differences between βTFP-deficient and wild-type mice (Fig. 3, Table 1). However, multivariable linear regression analysis incorporating age and sex as covariates demonstrated that SV was significantly associated with both age and sex, whereas CO showed a significant positive association with age. After adjustment, genotype-dependent differences in SV and CO were no longer significant, and no genotype-by-age interactions were observed (Table S1).

Sex-specific differences were observed in ventricular volumes. Within the homozygous βTFP-deficient group, one-way ANOVA with Tukey's post hoc testing demonstrated higher left ventricular end-diastolic volume (LVEDV) in males compared with that in females, whereas LVESV did not differ significantly by one-way ANOVA with Tukey's post hoc test (Fig. 3, Table 1). Direct comparison between sexes using an unpaired two-tailed *t*-test confirmed higher LVEDV and LVESV in homozygous males compared with those in homozygous females. Multivariable analysis further demonstrated that LVEDV was primarily associated with sex, whereas LVESV was influenced by both sex and genotype, with higher values observed in homozygous βTFP-deficient mice. Age was not significantly associated with ventricular volumes, and inclusion of age as a covariate did not materially alter these findings (Table S1).

### Impaired systolic deformation in βTFP-deficient mice

#### Representative strain patterns demonstrate altered systolic deformation

Representative circumferential, radial and longitudinal strain maps illustrate systolic myocardial deformation in wild-type and βTFP-deficient mice of both sexes (Fig. 4A-L). Compared with wild-type animals, βTFP-deficient mice exhibited visibly reduced myocardial deformation, most prominently in the longitudinal direction, reflected by attenuation of peak systolic strain magnitude in both males and females.

**Fig. 4.**
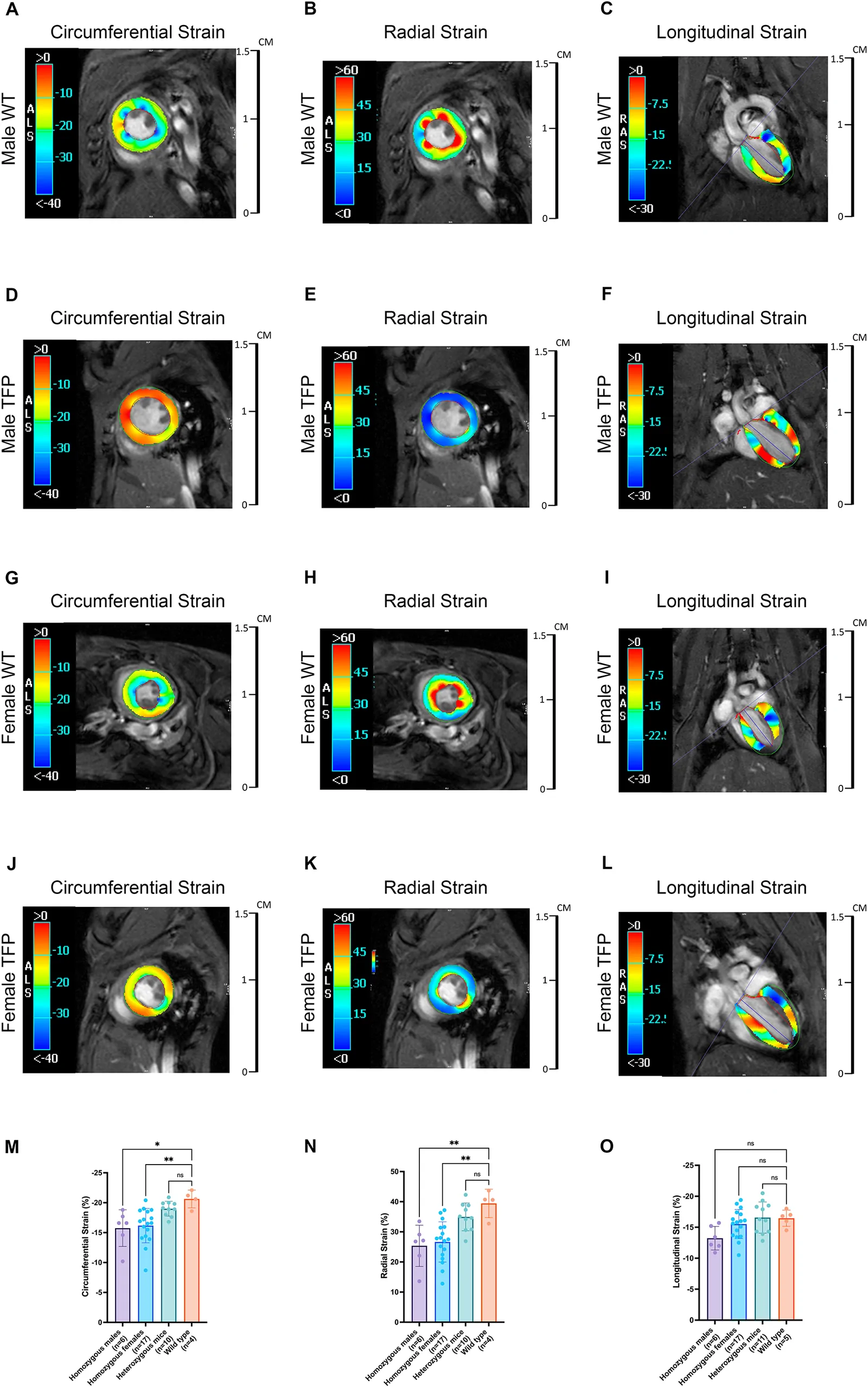
**Systolic myocardial deformation assessed by CMR strain analysis.** (A-L) Representative global peak systolic strain maps obtained using cardiac magnetic resonance imaging (CMR) illustrate circumferential, radial, and longitudinal deformation patterns in wild-type (WT) and βTFP-deficient mice. Images are from a WT male (A-C), homozygous βTFP-deficient male (D-F), WT female (G-I) and homozygous βTFP-deficient female (J-L). Pixel color represents the magnitude and direction of myocardial strain. In WT mice, circumferential and longitudinal strains show predominantly negative values (blue), whereas radial strain shows positive values (red), consistent with normal systolic deformation. In βTFP-deficient mice, circumferential and longitudinal strain maps display regions of reduced deformation, indicated by a shift toward warmer colors (yellow to red), whereas radial strain maps show areas of reduced positive deformation, indicated by cooler colors (light to deep blue). (M-O) Quantitative analysis of global peak systolic strain parameters: circumferential strain (M), radial strain (N) and longitudinal strain (O). For graphical representation, negative circumferential and longitudinal strain values are displayed on an upward scale, such that increasingly negative values (i.e. greater strain magnitude) are represented by taller bars. Data are shown for WT and heterozygous, and homozygous βTFP-deficient mice; homozygous mice are shown separately by sex, whereas WT and heterozygous mice are shown as combined groups. Data points indicate individual animals, with bars representing mean±s.d. Statistical comparisons among groups were performed using one-way ANOVA followed by Dunnett's or Šídák's multiple comparisons testing, as appropriate, with WT mice as the reference group. ns, not significant, *P*>0.05; *P≤0.05; **P≤0.01. Scale bars on the right of A-L are in cm. Color scales on the left indicate the directional orientations. ALS, anterior, left, superior; RAS, right, anterior, superior.

#### Quantitative analysis of global peak systolic strain parameters

Quantitative analysis demonstrated direction-specific alterations in systolic myocardial deformation in βTFP-deficient mice. One-way ANOVA with Dunnett's or Šídák's post hoc testing, as appropriate, showed differences in peak systolic strain parameters across groups, with significant reductions in circumferential and radial strain in homozygous mice and a trend toward reduced longitudinal strain, particularly in males (Fig. 4M-O, Table 2).

**Table 2.** CMR-derived myocardial deformation and extracellular volume parameters in wild-type and βTFP-deficient mice

Mouse group	Circumferential strain (%)	Radial strain (%)	Longitudinal strain (%)	ECV
Wild type	−20.63±1.48	39.43±4.71	−16.46±1.31	0.47±0.10
Homozygous males	−15.75±3.06 (0.0126)	25.37±6.85 (0.0026)	−13.23±1.90 (0.0536)	0.55±0.10 (0.4624)
Homozygous females	−16.17±2.87 (0.0077)	26.65±6.68 (0.0015)	−15.52±2.34 (0.7036)	0.52±0.06 (0.7396)
Heterozygous	−18.99±1.24 (0.6087)	34.96±4.62 (0.4078)	−16.56±2.52 (0.9994)	0.46±0.15 (0.9975)

Extracellular volume (ECV) fraction was derived from pre- and post-contrast T1 mapping as described in the Materials and Methods. Groups: homozygous βTFP-deficient mice are shown separately by sex; wild-type and heterozygous βTFP-deficient mice are shown as combined groups. Sample sizes: wild type, *n*=5; heterozygous, *n*=11; homozygous males, *n*=6; homozygous females, *n*=17; with the following exceptions. For circumferential and radial strain analyses, one wild-type and one heterozygous mouse were excluded following identification as outliers by Grubbs' test (α=0.05). Statistics: data are presented as mean±s.d., with *P*-values in parentheses. Statistical comparisons were performed using one-way ANOVA followed by multiple comparisons tests (Dunnett's or Šídák's, as appropriate) with wild-type mice as the reference group.

Multivariable linear regression analysis provided further refinement of these findings. Longitudinal strain was significantly associated with genotype and sex, with reduced deformation observed in homozygous mice and in males. In contrast, circumferential and radial strain were not significantly associated with genotype, sex or age in primary analyses. Sensitivity analyses indicated that these parameters were influenced by individual data points, including outliers, and robust regression suggested a genotype-dependent reduction; however, these effects were less consistent than those observed for longitudinal strain (Table S1). Overall, these findings indicate robust impairment of longitudinal myocardial deformation, with more variable and less consistent alterations in circumferential and radial strain.

Heterozygous βTFP-deficient mice did not demonstrate consistent differences in peak systolic strain parameters compared with wild-type mice across analyses (Fig. 4M-O, Table 2).

### Tissue characterization by CMR-derived ECV

#### Representative myocardial ECV maps demonstrate increased fibrosis

Representative myocardial extracellular volume (ECV) color maps illustrate myocardial tissue characteristics in wild-type and βTFP-deficient mice of both sexes (Fig. 5A-D). Homozygous βTFP-deficient mice demonstrated regions of increased myocardial ECV, visualized as red-colored areas, consistent with diffuse interstitial fibrosis and in agreement with histological findings.

**Fig. 5.**
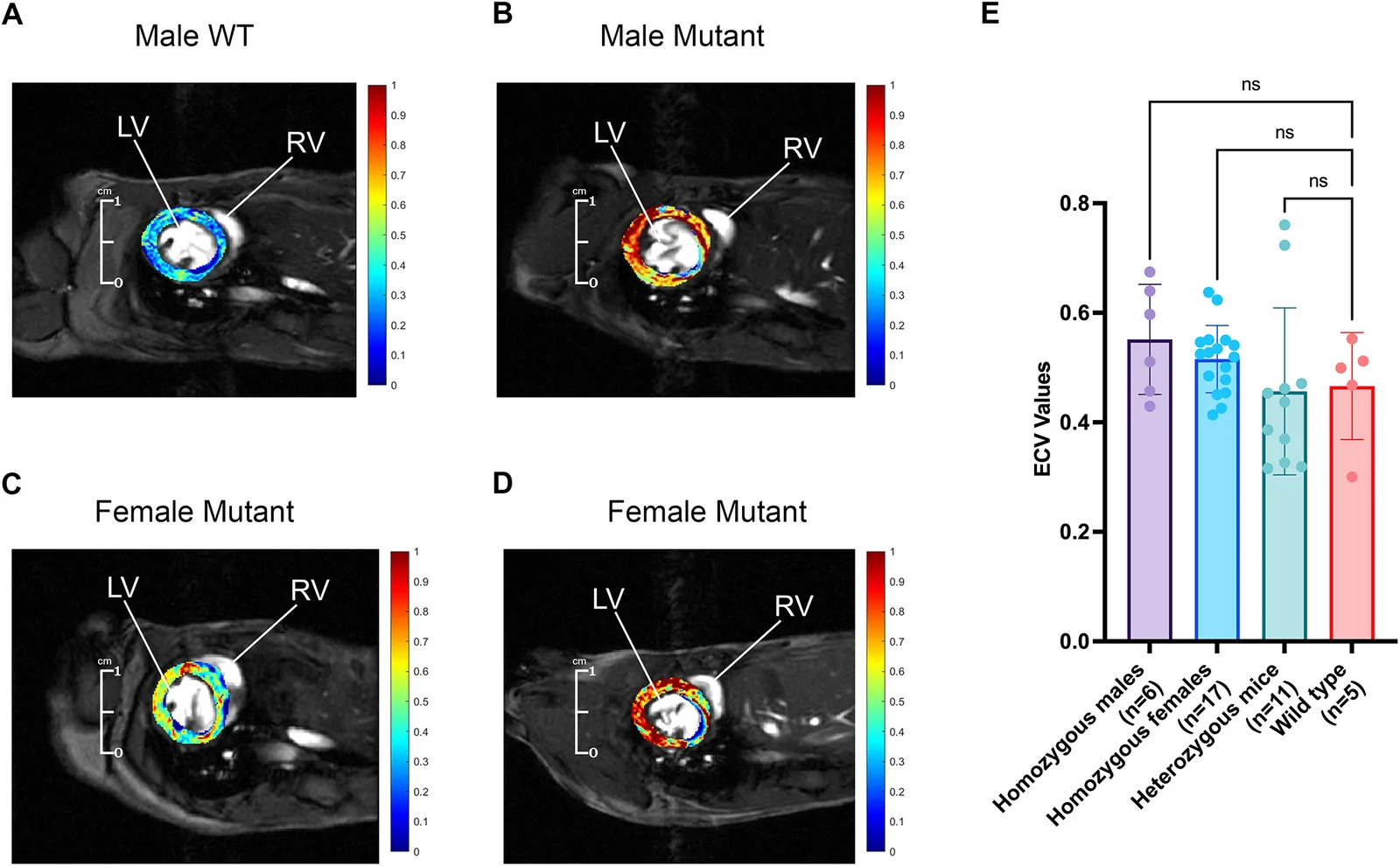
**Myocardial tissue characterization by CMR-derived extracellular volume.** (A-D) Representative myocardial extracellular volume (ECV) color maps illustrating myocardial tissue characteristics in wild-type (WT) and βTFP-deficient mice of both sexes: WT male (A), homozygous βTFP-deficient male (B), WT female (C) and homozygous βTFP-deficient female (D). Regions of increased ECV are visualized as red-colored areas, consistent with myocardial extracellular expansion. LV, left ventricle; RV, right ventricle. (E) Quantitative analysis of myocardial ECV. Data are shown for WT and heterozygous, and homozygous βTFP-deficient mice; homozygous mice are shown separately by sex, whereas wild-type and heterozygous mice are shown as combined groups. Data points indicate individual animals, with bars representing mean±s.d. Statistical comparisons among groups were performed using one-way ANOVA followed by Šídák's multiple-comparisons testing with WT mice as the reference group. ns, not significant, *P*>0.05. Scale bars on the left of A-D are in cm.

#### Quantitative analysis of myocardial ECV

Quantitative analysis of myocardial ECV using one-way ANOVA with Šídák's post hoc testing with wild-type mice as the reference group did not demonstrate statistically significant differences across groups (Fig. 5E, Table 2). Inspection of individual data points indicated variability in ECV measurements, particularly within the wild-type group. Consistent with this, multivariable linear regression analysis showed no significant association between genotype and ECV, and ECV values were not significantly associated with age or sex (Table S1). Despite the absence of statistically significant differences in quantitative ECV, the qualitative imaging findings and histological analyses consistently demonstrated increased myocardial fibrosis in homozygous βTFP-deficient mice.

### Reduced survival in homozygous βTFP-deficient mice with marked sex differences

Kaplan–Meier analysis demonstrated significantly reduced survival in homozygous male βTFP-deficient mice compared with that in homozygous females and heterozygous mice (Fig. 6; Mantel–Cox log-rank test, *P*=0.0003). Similar results were obtained using the Gehan–Breslow–Wilcoxon test (*P*<0.0001). These findings indicate increased vulnerability of homozygous males and align with the more severe cardiac structural and functional abnormalities observed in this group.

**Fig. 6.**
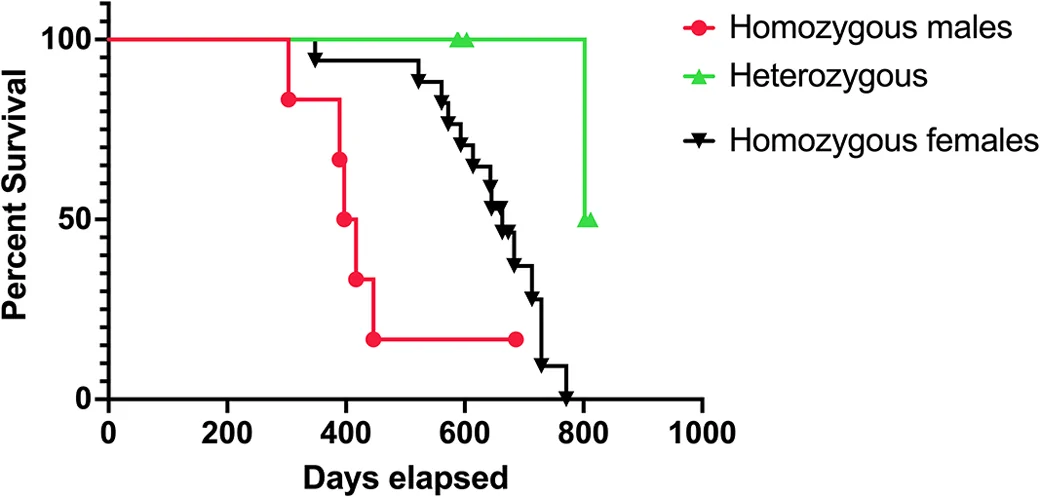
**Sex-dependent survival differences in βTFP-deficient mice.** Kaplan–Meier survival curves for homozygous βTFP-deficient males (*n*=6), homozygous βTFP-deficient females (*n*=17) and heterozygous βTFP-deficient mice (*n*=9; four males and five females). Statistical significance was assessed using the Mantel–Cox log-rank test (*P*=0.0003). Similar results were obtained using the Gehan–Breslow–Wilcoxon test (*P*<0.0001).

## DISCUSSION

In this study, we employed CMR to comprehensively characterize myocardial involvement in βTFP-deficient mice. Our findings demonstrate that cardiomyopathy in this model is associated with histologically confirmed myocardial fibrosis, supported by automated whole-slide collagen quantification, accompanied by reduced global systolic function and impaired systolic deformation. Multivariable analyses incorporating age and sex as covariates confirmed that key abnormalities, particularly reduced LVEF and increased LVESV, remained significantly associated with genotype after adjustment. Longitudinal strain impairment was also independently associated with genotype and sex, whereas alterations in circumferential and radial strain were less consistent across analyses. These structural and functional abnormalities were associated with reduced survival in homozygous animals. Notably, female βTFP-deficient mice exhibited milder cardiac dysfunction and prolonged survival compared with males, consistent with sex-dependent differences reported in other rodent models of heart disease (Wang et al., 2022). Although these findings suggest that cardiac involvement contributes to reduced survival, βTFP deficiency is a systemic disorder and additional metabolic or extra-cardiac factors may also influence outcomes. Together, these results identify fibrotic remodeling as a prominent feature of cardiac involvement in this model and establish a framework for future longitudinal and interventional studies in LC-FAODs.

Diffuse interstitial fibrosis quantified by CMR-derived ECV is an early, non-invasive marker of adverse outcomes across a wide spectrum of cardiac diseases, often preceding overt functional decline (Schelbert et al., 2015). ECV shows a strong correlation with histologically quantified diffuse myocardial fibrosis obtained from cardiac biopsies, supporting its validity as a surrogate marker of fibrotic burden (Chin et al., 2017). In clinical CMR, parametric ECV maps are commonly interpreted alongside global ECV measurements to capture spatial heterogeneity of myocardial fibrosis. In the present study, qualitative ECV mapping and histological analyses consistently supported the presence of myocardial fibrosis in homozygous βTFP-deficient mice, although quantitative ECV differences did not remain statistically significant in adjusted analyses. Myocardial fibrosis compromises the structural and functional integrity of the heart through multiple mechanisms, including impaired contractility, increased myocardial stiffness, cardiomyocyte slippage, electrical instability and reduced capillary density (Kong et al., 2014; Segura et al., 2014).

In LC-FAODs, cardiomyopathy has traditionally been attributed to a combination of intracellular lipid accumulation, impaired mitochondrial ATP production and the toxic effects of long-chain 3-hydroxyacylcarnitines (Cox, 2007; Merritt et al., 2020; Ramaccini et al., 2020; Tonin et al., 2013; Wasmus and Dudek, 2020). Although myocardial lipid profiling was not performed in the present study, βTFP-deficient mice in our colony exhibit systemic accumulation of long-chain acylcarnitines in plasma and fibroblast culture media (E.V.N., S.B., J.V. and C. Van't Land, unpublished observations from our laboratory). Such systemic metabolic perturbations may contribute to sustained myocardial metabolic stress and promote secondary remodeling, including fibrosis.

Beyond defining the structural and functional cardiac phenotype associated with βTFP deficiency, this study highlights the value of CMR for longitudinal assessment of cardiomyopathy in LC-FAODs. The ability to non-invasively assess myocardial fibrosis over time provides a unique opportunity to capture disease progression and pathological processes within a compressed timeframe, which, in humans, often unfold over years or decades. Importantly, CMR-derived tissue characterization can be directly compared with histological analyses, strengthening its translational relevance. In this context, myocardial fibrosis likely contributes to left ventricular decompensation in βTFP-deficient mice. Fibrosis represents an especially attractive therapeutic target, as interventions aimed at improving mitochondrial function and cellular energetics have already demonstrated beneficial effects on cardiac performance in patients with metabolic inherited disorders, including LC-FAODs (Hornby et al., 2022; Vockley et al., 2016). Accordingly, the βTFP-deficient mouse model, combined with longitudinal CMR, provides a robust preclinical platform to evaluate therapies designed to prevent or attenuate the development of cardiac fibrosis in LC-FAODs.

Although cardiac structure and function have been evaluated in several FAO-deficient mouse models using echocardiography and, in some cases, magnetic resonance imaging (MRI) or magnetic resonance spectroscopy, *in vivo* characterization of myocardial tissue composition – particularly diffuse interstitial fibrosis using CMR-derived ECV – remains limited. In earlier studies, Cox et al. (2009) described cardiac hypertrophy in VLCAD- and LCAD (also known as ACADL)-deficient mice, assessed primarily by heart weight and echocardiography, without histological evaluation of myocardial fibrosis. In particular, the more pronounced hypertrophy observed in male LCAD-deficient mice is consistent with the sex-dependent differences identified in the present study. The marked hypertrophy observed in LCAD-deficient mice likely reflects the prominent role of this enzyme in rodent long-chain FAO, in contrast to human metabolism, in which VLCAD predominates (Houten and Wanders, 2010). Similarly, Exil et al. (2003) reported microvesicular lipid accumulation in the hearts of VLCAD-deficient mice but did not observe changes in heart weight or perform functional or fibrotic assessments.

Using MRI and positron emission tomography imaging, Tucci et al. (2014) demonstrated progressive systolic dysfunction and impaired myocardial energetics and glucose metabolism in aged VLCAD-deficient mice, further supporting the development of cardiomyopathy in LC-FAOD models. More recently, Gaston et al. (2023) generated a mouse model of TFPα subunit deficiency using CRISPR/Cas9-mediated introduction of the HADHA c.1528G>C (p.E510Q) variant and demonstrated reduced ejection fraction, lower CO and SV, and increased left ventricular mass in both male and female homozygous mice using echocardiography. However, myocardial fibrosis was not evaluated by histology or imaging. Notably, many of these earlier investigations predated the widespread application of multiparametric CMR and ECV mapping.

Although myocardial fibrosis was present in both sexes, male βTFP-deficient mice exhibited greater alterations in ventricular volumes and reduced survival compared with females, indicating that sex-dependent factors modulate the functional and prognostic consequences of fibrotic remodeling rather than its extent. Sex-specific differences in mitochondrial biology have been reported in TFP/LCHAD deficiency, including differences in cardiolipin remodeling and monolysocardiolipin accumulation in patient-derived cells and βTFP-deficient mice, underscoring the importance of considering sex as a biological variable in preclinical and translational studies (Vieira Neto et al., 2024, 2026). Although the mechanistic basis of these differences remains incompletely understood, our findings suggest that sex-dependent modifiers influence disease progression and outcomes in LC-FAOD-associated cardiomyopathy.

This study has several limitations. First, although myocardial fibrosis was confirmed by histopathological examination and automated whole-slide collagen quantification and was supported by qualitative CMR-derived ECV mapping, quantitative ECV measurements demonstrated variability across groups. Direct myocardial lipidomic or acylcarnitine profiling was not performed, limiting mechanistic insight into the molecular drivers of fibrotic remodeling. Systemic accumulation of long-chain acylcarnitines observed in βTFP-deficient mice suggests chronic metabolic stress, but tissue-specific contributions within the heart remain to be defined. Second, although CMR provides robust and reproducible assessment of cardiac structure, function and tissue characteristics, the study was not designed to establish causality between fibrosis and systolic dysfunction. Age differences across experimental groups also represented a potential confounding factor; therefore, multivariable linear regression analyses incorporating age and sex as covariates were performed. Although key findings remained significant after adjustment, residual confounding cannot be completely excluded. In addition, wild-type controls were obtained from a commercial vendor rather than generated as littermates, which may introduce environmental or facility-related variability. Finally, longitudinal CMR was not performed in the same animals. Future studies incorporating serial imaging will be important for defining the temporal relationships among metabolic stress, fibrosis progression and functional decline.

In conclusion, βTFP-deficient mice develop a cardiomyopathy characterized by myocardial fibrosis, impaired systolic deformation, reduced global systolic function and decreased survival. Using multiparametric CMR, we demonstrate structural, functional and tissue-level abnormalities that parallel histological evidence of myocardial remodeling. Although the mechanisms linking mitochondrial FAO defects to cardiac fibrosis remain incompletely understood, our findings identify fibrotic remodeling as an important component of cardiac involvement in this model. These results support the βTFP-deficient mouse as a clinically relevant preclinical model of cardiomyopathy in LC-FAODs and highlight the value of CMR for longitudinal assessment of disease progression and therapeutic response. The combination of non-invasive imaging and a well-characterized metabolic phenotype positions this model as a useful platform for mechanistic studies and for evaluating interventions targeting cardiac dysfunction and myocardial remodeling in LC-FAODs.

## MATERIALS AND METHODS

### Mice

Mice carrying the *Hadhb* c.1210T>A (p.Met404Lys) missense mutation (βTFP-deficient allele) on a C57BL/6J background were maintained and bred at Charles River Laboratories using an established breeding scheme. βTFP-deficient mice were generated by crossing homozygous males with heterozygous females, yielding homozygous and heterozygous offspring for experimental studies. Wild-type C57BL/6J mice were purchased from The Jackson Laboratory and used as controls. All animal procedures were approved by the University of Pittsburgh Institutional Animal Care and Use Committee (protocol 21090074) and conducted in accordance with National Institutes of Health guidelines for the care and use of laboratory animals. Genotyping was performed by Transnetyx (Cordova, TN, USA) using DNA samples collected by Charles River Laboratories. Allele-specific quantitative PCR assays were used to distinguish wild-type and mutant *Hadhb* alleles. The following primers were used: forward, 5′-CATGGATTCTGATTGGTTTGCACAA-3′, and reverse, 5′-TTCAAAATATTAACACGCTGGTAAATTCAAACATAT-3′. Two allele-specific probes were used, one targeting the wild-type sequence (5ʹ-TGGTTTTCCTACCCTTGTAGT-3ʹ) and one targeting the mutant sequence (5ʹ-TGGTTTTCCTACCCATGTAGT-3ʹ). Both male and female mice were included. Due to breeding constraints inherent to the model, age at imaging varied across genotypes. Ages of homozygous males ranged from 26 weeks 5 days to 30 weeks 5 days, whereas ages of homozygous females ranged from 14 weeks 0 days to 31 weeks 4 days. Ages of heterozygous mice ranged from 12 weeks 6 days to 28 weeks 1 day. Wild-type mice were age-matched within a narrow range (30 weeks 1 day to 30 weeks 2 days). Wild-type mice comprised three males and two females, whereas the heterozygous group comprised four males and seven females. Age was incorporated as a covariate in multivariable regression analyses, as described in the ‘Statistical analysis’ section.

### CMR

CMR was performed using a Bruker BioSpec 70/30 USR superconducting magnet system (7.0 Tesla) at the Rangos Research Center Animal Imaging Core, University of Pittsburgh, following established core protocols (Wu, 2021). Mice were anesthetized with isoflurane delivered in oxygen, and heart rate and respiration were continuously monitored throughout the imaging session. Body temperature was maintained using a heated animal support to minimize physiological variability during image acquisition. All image acquisition and analyses were performed by investigators masked to genotype.

#### Global systolic function

Global cardiac function was assessed using multi-slice cine MRI acquired in both long-axis and contiguous short-axis orientations covering the entire left ventricle from base to apex. Cine images were obtained with electrocardiographic and respiratory gating to minimize motion artifacts. LVEDV and LVESV were quantified by manual endocardial contouring, and LVEF, SV and CO were calculated using standard formulas.

#### Cardiac strain analysis

Myocardial strain analysis was performed using cine MRI images and analyzed with commercially available software (cvi42, Circle Cardiovascular Imaging, Calgary, Canada). Global circumferential, radial and longitudinal peak systolic strains were quantified using feature-tracking techniques applied to standard long-axis and short-axis cine images. Strain values are reported as peak systolic deformation and expressed as percentages.

#### Myocardial fibrosis quantification

Diffuse myocardial fibrosis was assessed non-invasively by quantification of myocardial ECV using T1 mapping acquired before and after administration of a clinical-grade gadolinium-based contrast agent, gadobenate dimeglumine (MultiHance®, Bracco Diagnostics, Inc., Monroe Township, NJ, USA). A bolus of gadolinium contrast with a dosage of 0.02 mmol/kg of body weight was administered via a subcutaneous catheter, and post-contrast T1 maps were acquired after sufficient equilibration time. Native and post-contrast T1 values were measured in both myocardium and blood pool regions of interest. ECV was calculated using the following equation: ECV=(1−hematocrit)×(ΔR1_myocardium_/ΔR1_blood_), where R1=1/T1 and ΔR1 represents the difference between post-contrast and pre-contrast R1 values.

### Histological analysis

Following euthanasia, hearts were harvested, fixed overnight in 10% neutral buffered formalin, processed, embedded in paraffin and sectioned at 5 μm thickness using an RM2255 microtome (Leica Biosystems, Deer Park, IL, USA). Sections were stained with Hematoxylin and Eosin for routine histological assessment and with PSR to evaluate myocardial interstitial fibrosis.

Histological analyses were performed in a separate cohort comprising eight homozygous βTFP-deficient mice (six males and two females). All histological processing and staining were performed by a commercial histopathology service (HistoWiz, New York, NY, USA). Histological evaluation was performed by a board-certified veterinary pathologist, masked to genotype. Myocardial fibrosis severity was assessed using a semi-quantitative five-point scoring system (0, within normal limits; 1, minimal; 2, mild; 3, moderate; 4, severe).

Quantitative collagen fraction analysis was performed on PSR-stained sections using HALO version 4.2.6399 (Indica Labs, Albuquerque, NM, USA) with the ‘Area Quantification’ algorithm (v3.0.1). Following manual definition of background and collagen staining thresholds, collagen-positive areas were automatically identified based on weak, moderate and strong staining intensities. Whole-slide analysis was performed with manual exclusion of obvious large blood vessels. The collagen fraction was calculated as the collagen-positive area divided by the total myocardial tissue area and expressed as a percentage. All image analyses were performed by an investigator masked to genotype.

### Statistical analysis

Statistical analyses were performed using Prism for macOS version 11.0.1 (GraphPad Software, Boston, MA, USA) and Stata/SE version 12.1 for Mac (StataCorp LP, College Station, TX, USA). Data are presented as mean±s.d. unless otherwise indicated. Outliers were identified using Grubbs' test (α=0.05) and excluded only from the specific analyses in which they were detected.

Group comparisons were performed using one-way ANOVA followed by Dunnett's or Šídák's multiple comparisons tests, as appropriate, with wild-type mice used as the reference group. Direct comparisons between homozygous male and female βTFP-deficient mice were performed using unpaired two-tailed *t*-tests where indicated.

Kaplan–Meier survival curves were analyzed using the Mantel–Cox (log-rank) test. Gehan–Breslow–Wilcoxon testing was performed as a sensitivity analysis.

To evaluate potential confounding by age, multivariable linear regression analyses were performed for key CMR outcomes, including LVEF, SV, CO, LVEDV, LVESV, myocardial strain parameters and ECV. Age (weeks) and sex were included as covariates. Secondary models additionally evaluated genotype-by-age interaction terms. Robust standard errors were used for all regression analyses. Results are summarized in Table S1.

A two-sided *P*-value <0.05 was considered statistically significant.

## Supplementary Material

10.1242/dmm.052921_sup1 Supplementary information
