# Supplementary material for: Cardiac magnetic resonance imaging reveals myocardial fibrosis and systolic dysfunction in mitochondrial trifunctional protein-deficient mice
Source: Dis Model Mech. 2026 Sep 25;19(9):dmm052921. doi: 10.1242/dmm.052921 (PMC13651953; doi:10.1242/dmm.052921)
Supplement: Supplementary file 1 [file dmm-19-052921-s1.pdf]

Table S1. Multivariable linear regression analyses of cardiac MRI parameters adjusted for genotype, sex, and age

Parameter	Genotype effect (Homozygous vs WT) P value	Sex effect P value	Age effect P value	Genotype × age interaction P value	Main interpretation
LVEF	0.002	0.100	0.019	NS	Genotype-dependent systolic dysfunction maintained after adjustment
SV	0.063	0.014	0.002	NS	Primarily associated with age and sex
CO	0.414	0.969	0.003	NS	Primarily associated with age
LVEDV	0.910	0.000	0.713	NS	Predominantly sex-associated
LVESV	0.044	0.003	0.513	NS	Associated with genotype and sex
Longitudinal strain	0.039	0.001	0.145	NS	Robust genotype-associated impairment
Circumferential strain	0.638	0.573	0.146	NS	Variable across analyses*
Radial strain	0.274	0.856	0.069	NS	Variable across analyses*
ECV	0.225	0.590	0.738	NS	No significant adjusted associations

Multivariable linear regression analyses included genotype, sex, age, and genotype-by-age interaction terms. Robust standard errors were used in all models. Genotype comparisons are shown for homozygous βTFP-deficient mice relative to wild-type controls. **Abbreviations:** LVEF, left ventricular ejection fraction; SV, stroke volume; CO, cardiac output; LVESV, left ventricular end-systolic volume; LVEDV, left ventricular end-diastolic volume; ECV, extracellular volume; NS, non-significant.

*Robust regression sensitivity analyses were additionally performed for circumferential and radial strain parameters due to the influence of outliers.
